# Inpatient outcomes after primary total knee arthroplasty in patients receiving chronic dialysis: A nationwide propensity score-matched analysis

**DOI:** 10.1371/journal.pone.0358078

**Published:** 2026-09-15

**Authors:** Ela Cohen Nissan, Yaara Berkovich, David Maman, Yaniv Steinfeld, Yaron Berkovich

**Affiliations:** 1 Technion – Israel Institute of Technology, The Ruth and Bruce Rappaport Faculty of Medicine, Technion City, Haifa 3200003, Israel; 2 Department of Orthopedics, Carmel Medical Center, Haifa, Israel; International University of Health and Welfare, School of Medicine, JAPAN

## Abstract

**Background:**

Total knee arthroplasty (TKA) is widely performed for end-stage knee osteoarthritis, but outcomes may be less favorable in medically complex patients. Patients receiving chronic dialysis represent a particularly high-risk population because of chronic kidney disease-mineral and bone disorder, impaired physiologic reserve, and substantial systemic comorbidity. This study evaluated inpatient outcomes, hospital resource use, and in-hospital mortality after primary TKA among patients receiving chronic dialysis using a contemporary national inpatient database.

**Methods:**

Adult patients who underwent primary TKA between 2016 and 2022 were identified in the Nationwide Inpatient Sample. Chronic dialysis status was defined using ICD-10-CM code Z99.2. Primary outcomes were length of stay, total hospital charges, and in-hospital mortality. Secondary outcomes included in-hospital postoperative complications. To reduce baseline differences between groups, 10:1 propensity score matching was performed using demographic, surgical, and comorbidity variables. Covariate balance was assessed using standardized mean differences.

**Results:**

Among 3,423,989 patients who underwent primary TKA, 4,790 (0.1%) were receiving chronic dialysis. Before matching, patients receiving chronic dialysis had longer hospital stays, greater hospital charges, and higher in-hospital mortality than patients not receiving chronic dialysis. After matching, chronic dialysis remained associated with longer length of stay (4.2 vs. 2.7 days), higher total hospital charges, and increased risks of several in-hospital complications, including sepsis and in-hospital death. Because the Nationwide Inpatient Sample captures inpatient hospitalizations only, these findings should be interpreted as inpatient outcomes rather than post-discharge or long-term arthroplasty outcomes.

**Conclusions:**

Patients receiving chronic dialysis experienced substantially worse inpatient outcomes after primary TKA, even after adjustment for measured baseline differences. These findings support careful preoperative counseling, perioperative optimization, and multidisciplinary inpatient management when TKA is considered in this high-risk population.

**Levels of evidence:**

Level III – Retrospective Cohort Study.

## Introduction

Chronic kidney disease (CKD) is a common chronic condition that places a major burden on healthcare systems worldwide [1–7]. As CKD progresses to kidney failure requiring chronic dialysis, renal replacement therapy, most commonly hemodialysis or peritoneal dialysis, becomes necessary to maintain survival and quality of life [8–14]. In addition to systemic comorbidity, advanced kidney disease is associated with important musculoskeletal consequences, particularly chronic kidney disease-mineral and bone disorder, altered bone turnover, osteomalacia, and impaired bone quality, which may increase orthopaedic complexity [15–19]. In accordance with contemporary kidney disease nomenclature, we refer to the study population as patients receiving chronic dialysis rather than using older terminology such as end-stage kidney disease when possible.

Total knee arthroplasty (TKA) is one of the most commonly performed procedures for advanced knee osteoarthritis and is generally associated with substantial functional improvement [20–24]. However, performing TKA in patients who are dependent on dialysis may be especially challenging because these patients often have reduced physiologic reserve, multiple comorbidities, impaired bone quality, and increased susceptibility to infection and other perioperative complications [18,19,25–28].

Previous studies have suggested that patients receiving chronic dialysis may have worse outcomes after TKA, including higher complication rates and mortality [25–28]. A prior NIS-based study evaluated dialysis patients undergoing TKA during an earlier period and reported increased perioperative complications, length of stay, and hospital charges [25]. The present study adds to that literature by extending the analysis through 2022, evaluating a larger contemporary cohort, applying 10:1 propensity score matching, and reporting matched inpatient outcomes with risk estimates. Therefore, the aim of the present study was to evaluate in-hospital complications, in-hospital mortality, length of stay, and hospital charges after primary TKA in patients receiving chronic dialysis using the NIS. We hypothesized that patients receiving chronic dialysis would experience higher inpatient complication rates, greater hospital resource use, longer length of stay, and increased in-hospital mortality compared with patients not receiving chronic dialysis.

## Materials and methods

### Data source

This retrospective cohort study used data from the Nationwide Inpatient Sample (NIS), part of the Healthcare Cost and Utilization Project (HCUP). The NIS is a stratified, all-payer inpatient discharge database that includes information on hospitalizations from participating U.S. hospitals. Discharges between January 1, 2016, and December 31, 2022, were included.

The NIS has a complex survey design, and nationally representative estimates require use of discharge-level weights, hospital strata, and clustering variables. In the present analysis, discharge weights were used to generate national estimates. Survey design elements were considered when estimating national counts and proportions. Because the main comparative analysis used propensity score matching at the discharge level, matched-group comparisons are presented as inpatient discharge-level estimates. Therefore, all findings should be interpreted as hospitalization-based outcomes rather than patient-level longitudinal outcomes.

### Study population and patient identification

Adult patients aged 18 years or older who underwent primary TKA were identified using ICD-10-PCS procedure codes. Hospitalizations involving revision arthroplasty procedures, emergent or non-elective admissions, trauma-related admissions, malignancy-related admissions when applicable, and records with missing key demographic data were excluded. To reduce potential confounding related to perioperative outcomes during the pandemic period, hospitalizations associated with COVID-19 were also excluded.

Chronic dialysis status was defined using ICD-10-CM code Z99.2, which indicates dependence on renal dialysis. Patients were divided into two groups: those receiving chronic dialysis and those not receiving chronic dialysis. Because the NIS contains diagnosis and procedure codes from a single hospitalization and does not provide outpatient lookback information, dialysis duration, dialysis schedule, dialysis modality, or preoperative laboratory values, we could not distinguish hemodialysis from peritoneal dialysis, determine dialysis vintage, or verify dialysis timing relative to surgery. The use of Z99.2 was intended to identify patients with established chronic dialysis dependence rather than temporary dialysis for acute kidney injury; however, misclassification remains possible and is acknowledged as a limitation. The ICD-10-CM and ICD-10-PCS codes used to define primary TKA, exclusion criteria, chronic dialysis status, COVID-19 exclusion, comorbidities, and in-hospital postoperative complications are provided in S2 Table.

### Covariates and comorbidities

Patient characteristics included age, sex, race, and primary payer. Clinical comorbidities were identified using ICD-10-CM diagnosis codes and included dyslipidemia, chronic anemia, osteoporosis, alcohol abuse, type 2 diabetes, congestive heart failure, chronic lung disease, liver disease, prior myocardial infarction, prior cerebrovascular accident, and obesity. Use of robotic-assisted TKA was also recorded.

Definitions of comorbidities and outcomes were based on ICD-10-CM diagnosis codes recorded during the index hospitalization. Comorbidities were identified from diagnosis fields available in the NIS for that admission. Because the NIS does not include outpatient or prior-year claims, comorbidities reflect coded conditions during the surgical hospitalization and may not capture all historical diagnoses. Similarly, postoperative complications were defined as in-hospital coded events during the index admission only. Events occurring after discharge or during subsequent admissions were not captured.

### Outcomes of interest

The primary outcomes were inpatient length of stay (LOS), total hospital charges, and in-hospital mortality during the index TKA admission. Total hospital charges were obtained from the NIS total charge variable and represent hospital billing charges rather than actual costs or reimbursements. Therefore, the term “hospital charges” is used consistently throughout the manuscript.

Secondary outcomes included in-hospital postoperative complications recorded during the index admission, including blood loss anemia, urinary tract infection, intraoperative fracture, blood transfusion, ileus, pneumonia, sepsis, pulmonary embolism, and deep vein thrombosis. Because the NIS does not track patients longitudinally after discharge, these outcomes should be interpreted as inpatient complications only.

### Propensity score matching

Because patients receiving chronic dialysis differed substantially from patients not receiving chronic dialysis at baseline, 10:1 propensity score matching was performed to improve comparability between groups. Propensity scores were estimated using logistic regression with chronic dialysis status as the dependent variable. The matching model included age, sex, robotic-assisted surgery status, and comorbidities including dyslipidemia, chronic anemia, osteoporosis, alcohol abuse, type 2 diabetes, congestive heart failure, chronic lung disease, liver disease, prior myocardial infarction, prior cerebrovascular accident, and obesity. No hospital-level variables were included in the final propensity-score matching model.

Each patient receiving chronic dialysis was matched to up to 10 patients not receiving chronic dialysis using nearest-neighbor matching without replacement and a caliper width of 0.2 of the standard deviation of the logit of the propensity score. Covariate balance before and after matching was assessed using standardized mean differences (SMDs), with an absolute SMD < 0.10 considered indicative of acceptable balance. Detailed pre- and post-matching SMD values are provided in S1 Table.

### Statistical analysis

Continuous variables were summarized as mean ± SD and compared using independent-samples t-tests or survey-adjusted methods when appropriate. Categorical variables were summarized as counts and percentages and compared using chi-square tests. After propensity-score matching, outcome comparisons were performed between the matched dialysis and non-dialysis cohorts using standard group-based statistical tests. Matched-set clustering and robust variance estimation were not applied. For binary outcomes, absolute risks, risk differences, risk ratios, and 95% confidence intervals were calculated to improve clinical interpretability, particularly for rare outcomes such as sepsis and in-hospital mortality. A two-sided p value < 0.05 was considered statistically significant. Temporal trends in the proportion of TKA hospitalizations involving patients receiving chronic dialysis were assessed using logistic regression, with chronic dialysis status as the dependent variable and calendar year as a continuous independent variable. The corresponding p value was reported as the p value for trend.

Because very large administrative datasets can produce statistically significant p values for small absolute differences, interpretation emphasized effect sizes, absolute risks, risk differences, and standardized mean differences rather than p values alone. Propensity score matching was performed using MATLAB, and statistical analyses were performed using SPSS.

### Ethical aspects

This study used the HCUP Nationwide Inpatient Sample, a publicly available, de-identified administrative database. No direct patient contact occurred, and no identifiable private health information was accessed. Therefore, this study was exempt from institutional review board approval, and informed consent was not required.

## Results

Table 1 summarizes the demographic and clinical characteristics of all 3,423,989 total TKA patients, of whom 4,790 (0.1%) were receiving chronic dialysis. Patients with chronic dialysis status were slightly younger than those without (66.1 vs. 66.9 years, p < 0.01) and had a markedly lower proportion of female patients (46.8% vs. 61.5%, p < 0.01).

**Table 1:** Demographic and clinical characteristics of patients with and without chronic dialysis.

Parameter	No chronic dialysis	Chronic dialysis	Significance
Total Surgeries	3,419,299 (99.9%)	4,790 (0.1%)	–
Average Age (y)	66.9	66.1	P < 0.01
Female (%)	61.5	46.8	P < 0.01
Robotic Surgery (%)	5.1	3.3	P < 0.01
Dyslipidemia (%)	47.4	54.4	P < 0.01
Chronic Anemia (%)	5.8	12.1	P < 0.01
Osteoporosis (%)	4.1	3.3	P < 0.01
Alcohol abuse (%)	0.9	1.1	P = 0.07
Type 2 Diabetes (%)	21.8	53.5	P < 0.01
Congestive Heart Failure (%)	1.3	10	P < 0.01
Chronic Lung Disease (%)	6.1	11.4	P < 0.01
Liver Disease (%)	1.2	3.2	P < 0.01
History of Myocardial Infarction (%)	3.2	6.8	P < 0.01
History of Cerebrovascular Accident (%)	4.2	9.2	P < 0.01
Obesity (%)	31.8	31.7	P = 0.93

Robotic-assisted surgery was less frequently performed in the dialysis group (3.3% vs. 5.1%, p < 0.01). Several comorbidities were significantly more prevalent in patients receiving chronic dialysis, including dyslipidemia (54.4% vs. 47.4%), chronic anemia (12.1% vs. 5.8%), congestive heart failure (10% vs. 1.3%), chronic lung disease (11.4% vs. 6.1%), liver disease (3.2% vs. 1.2%), and history of myocardial infarction (6.8% vs. 3.2%) and cerebrovascular accident (9.2% vs. 4.2%) (p < 0.01 for all).

There were no significant differences between the groups regarding obesity (31.7% vs. 31.8%, p = 0.93).

### Trend in the prevalence of chronic dialysis among TKA patients (2016–2022)

**Fig 1:**
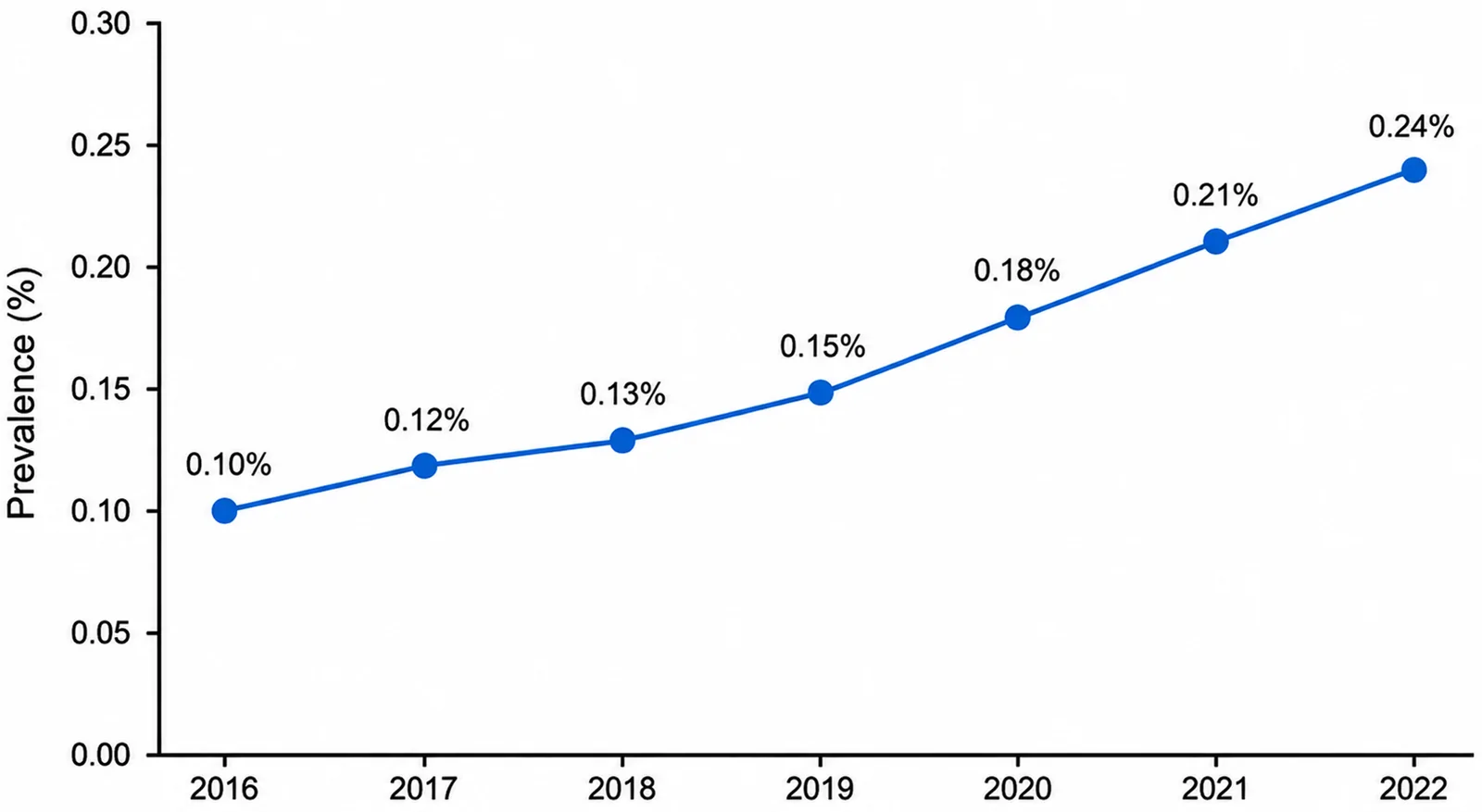
Trend in the prevalence of chronic dialysis among TKA patients, 2016-2022.

Fig 1 illustrates the yearly proportion of TKA patients receiving chronic dialysis between 2016 and 2022. The prevalence of chronic dialysis status increased steadily over the study period, rising from 0.10% in 2016 to 0.24% in 2022. This upward trend was statistically significant (p < 0.01), indicating a consistent growth in the proportion of patients receiving chronic dialysis undergoing TKA over time.

### Length of stay and total hospital charges in patients with and without chronic dialysis

Table 2 compares hospitalization outcomes between TKA patients with and without chronic dialysis. Patients undergoing dialysis had a significantly longer mean length of stay compared with those without chronic dialysis status (4.2 ± 3.7 vs. 2.3 ± 1.8 days, p < 0.01), representing an 82.6% increase in hospitalization duration.

**Table 2:** Length of stay and total hospital charges in patients with and without chronic dialysis.

	No chronic dialysis	Chronic dialysis	Percent Increase	Significance
Length of stay mean in days	2.3 (SD 1.8)	4.2 (SD 3.7)	82.6%	P < 0.01
Total charges mean in $	65,552 (SD 45,753)	99,668 (SD 81,426)	52.0%	P < 0.01

The mean total hospital charges were substantially higher in the dialysis group ($99,668 ± $81,426 vs. $65,552 ± $45,753, p < 0.01), reflecting a 52.0% increase in hospital charges compared with non-dialysis patients.

### In-hospital mortality in patients with and without chronic dialysis

Table 3 demonstrates that in-hospital mortality was markedly higher among TKA patients receiving chronic dialysis compared with those not receiving chronic dialysis (0.73% vs. 0.03%, p < 0.01). This corresponds to a 23.6-fold increased risk of death (95% CI: 16.86–33.14).

**Table 3:** In-hospital mortality in patients with and without chronic dialysis.

	No chronic dialysis	Chronic dialysis	Risk Ratio (95% CI)	Significance
Died during hospitalization in %	0.03%	0.73%	23.64(95% CI: 16.86–33.14)	P < 0.01

### Demographic and clinical characteristics after 10:1 propensity score matching

To address baseline differences and minimize potential selection bias, a 10:1 propensity score-matched analysis was conducted. Patients with and without chronic dialysis were matched based on key demographic and clinical variables, including age, sex, and major comorbidities.

After matching, the two groups were well balanced across measured characteristics (Table 4). Standardized mean differences were assessed as part of the matching diagnostics and supported acceptable post-matching balance. There were no statistically significant differences in age (66.2 vs. 66.1 years, p = 0.51), female proportion (46.9% vs. 46.8%, p = 0.82), or robotic surgery rates (3.1% vs. 3.3%, p = 0.36). Similarly, the prevalence of common comorbidities including dyslipidemia, chronic anemia, type 2 diabetes, congestive heart failure, chronic lung disease, liver disease, and history of myocardial infarction or cerebrovascular accident was comparable between groups (p > 0.05 for all).

**Table 4:** Demographic and clinical characteristics after 10:1 propensity score matching.

Parameter	No chronic dialysis	Chronic dialysis	Significance
Total Surgeries	47898 (90.9%)	4,790 (9.1%)	–
Average Age (y)	66.2	66.1	P = 0.51
Female (%)	46.9	46.8	P = 0.82
Robotic Surgery (%)	3.1	3.3	P = 0.36
Dyslipidemia (%)	55.2	54.4	P = 0.30
Chronic Anemia (%)	12.5	12.1	P = 0.43
Osteoporosis (%)	2.9	3.3	P = 0.12
Alcohol abuse (%)	1.3	1.1	P = 0.36
Type 2 Diabetes (%)	54.1	53.5	P = 0.45
Congestive Heart Failure (%)	9.6	10	P = 0.29
Chronic Lung Disease (%)	11.4	11.4	P = 0.88
Liver Disease (%)	3.1	3.2	P = 0.92
History of Myocardial Infarction (%)	6.9	6.8	P = 0.85
History of Cerebrovascular Accident (%)	9	9.2	P = 0.73
Obesity (%)	31.9	31.7	P = 0.81

These findings support improved comparability between groups after matching and provide the basis for subsequent matched outcome comparisons. All post-matching standardized mean differences were below 0.10, supporting adequate covariate balance between the matched dialysis and non-dialysis cohorts. Detailed SMD values before and after matching are presented in S1 Table.

### Length of stay and total hospital charges after 10:1 propensity score matching

Following 10:1 propensity score matching, patients receiving chronic dialysis continued to experience significantly greater inpatient resource utilization compared with matched controls (Table 5). Mean length of stay remained longer in the chronic dialysis group (4.2 ± 3.7 vs. 2.7 ± 1.7 days, p < 0.01), representing a 55.6% increase. Similarly, mean total hospital charges were higher among patients receiving chronic dialysis ($99,668 ± $81,426 vs. $62,369 ± $38,694, p < 0.01), representing a 59.8% increase.

**Table 5:** Length of stay and total hospital charges after 10:1 propensity score matching.

	No chronic dialysis	Chronic dialysis	Percent Increase	Significance
Length of stay mean in days	2.7 (SD 1.7)	4.2 (SD 3.7)	55.6%	P < 0.01
Total charges mean in $	62,369 (SD 38,694)	99,668 (SD 81,426)	59.8%	P < 0.01

### Postoperative thromboembolic complications after 10:1 propensity score matching

Table 6 compares the rates of thromboembolic events between matched cohorts. Pulmonary embolism was significantly less common among patients receiving chronic dialysis compared with their matched controls (0.1% vs. 0.3%, p = 0.01).

**Table 6:** Postoperative thromboembolic complications after 10:1 propensity score matching.

Parameter	No chronic dialysis	Chronic dialysis	Significance
Pulmonary embolism (%)	0.3	0.1	P = 0.01
DVT (%)	0.4	0.5	P = 0.12

The incidence of deep vein thrombosis (DVT) was slightly higher in the dialysis group (0.5% vs. 0.4%), but this difference did not reach statistical significance (p = 0.12).

### Postoperative complications in patients with and without chronic dialysis after 10:1 propensity score matching

**Fig 2:**
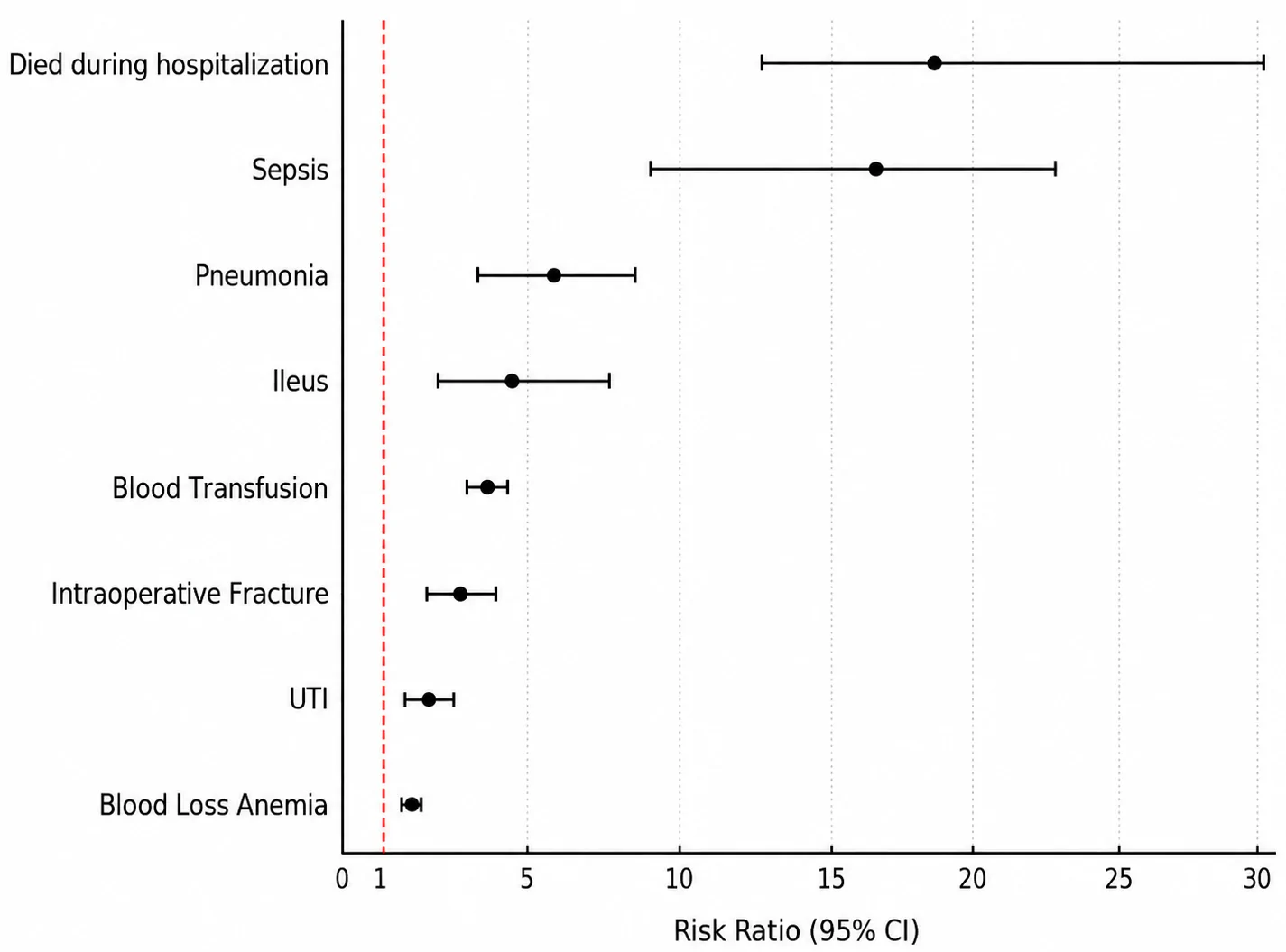
Forest plot of in-hospital postoperative complications in patients with and without chronic dialysis after 10:1 propensity score matching.

Table 7 and Fig 2 present event counts, denominators, absolute risks, risk differences, and risk ratios with 95% confidence intervals for major in-hospital postoperative complications among TKA patients receiving chronic dialysis compared with 10:1 propensity score-matched controls not receiving chronic dialysis. Even after rigorous adjustment for baseline differences, chronic dialysis status remained strongly associated with worse outcomes. Significantly increased risks were observed for blood loss anemia (RR 1.5, 95% CI 1.4–1.6), urinary tract infection (RR 1.8, 95% CI 1.4–2.2), intraoperative fracture (RR 2.4, 95% CI 1.8–3.2), blood transfusion (RR 3.5, 95% CI 3.2–4.0), ileus (RR 4.0, 95% CI 2.4–6.7), and pneumonia (RR 5.0, 95% CI 3.5–7.2) (p < 0.01 for all). The most pronounced effects were observed for sepsis (RR 13.4, 95% CI 8.3–21.6) and in-hospital mortality (RR 17.6, 95% CI 10.2–30.5). Because several outcomes were rare, absolute risks and risk differences were also evaluated. Although relative risks were high for outcomes such as sepsis and in-hospital mortality, the absolute event rates remained low. Therefore, these findings should be interpreted as clinically important increases in inpatient risk, but not as estimates of long-term mortality or post-discharge complication risk.

**Table 7:** Event counts, absolute risks, risk differences, and risk ratios for major in-hospital outcomes after 10:1 propensity score matching. Values represent weighted national discharge estimates after propensity-score matching. Risk differences are expressed as percentage-point differences between the chronic dialysis and non-dialysis cohorts.

Outcome	No chronic dialysis events/ denominator	No chronic dialysis risk	Chronic dialysis events/ denominator	Chronic dialysis risk	Risk difference	Risk ratio (95% CI)
Blood loss anemia	7,670/ 47,898	16.013%	1,080/ 4,790	22.547%	6.534%	1.41 (1.33–1.49)
Urinary tract infection	630/ 47,898	1.315%	110/ 4,790	2.296%	0.981%	1.75 (1.43–2.13)
Intraoperative fracture	250/ 47,898	0.522%	60/ 4,790	1.253%	0.731%	2.40 (1.81–3.18)
Blood transfusion	1,330/ 47,898	2.777%	440/ 4,790	9.186%	6.409%	3.31 (2.98–3.67)
Ileus	50/ 47,898	0.104%	20/ 4,790	0.418%	0.313%	4.00 (2.38–6.71)
Pneumonia	90/ 47,898	0.188%	45/ 4,790	0.939%	0.752%	5.00 (3.50–7.14)
Sepsis	30/ 47,898	0.063%	40/ 4,790	0.835%	0.772%	13.33 (8.31–21.39)
In-hospital mortality	20/ 47,838	0.042%	35/ 4,785	0.731%	0.690%	17.50 (10.11–30.28)

## Discussion

In this nationwide inpatient analysis of primary TKA, patients receiving chronic dialysis experienced substantially worse inpatient outcomes than patients not receiving chronic dialysis. Even after 10:1 propensity score matching, chronic dialysis was associated with longer hospital stays, higher hospital charges, and markedly increased risks of several serious in-hospital complications, including sepsis and in-hospital mortality. These findings support chronic dialysis status as an important marker of inpatient perioperative risk among patients undergoing primary TKA.

Patients receiving dialysis are known to have impaired bone quality and a high burden of systemic disease [18,19,25–28]. This likely contributes to the increased perioperative vulnerability observed in our cohort. The elevated risks of blood transfusion, pneumonia, ileus, urinary tract infection, intraoperative fracture, sepsis, and mortality observed in the present study are broadly consistent with prior reports describing poorer arthroplasty outcomes in patients receiving chronic dialysis [18,25,27–30]. Our results extend this literature by providing a large contemporary national analysis showing that these associations persist even after matching for measured baseline differences.

Another important finding was the substantially higher resource utilization among patients receiving chronic dialysis. Both LOS and total hospital charges remained significantly higher after matching. These differences likely reflect the complexity of perioperative management in patients with kidney failure requiring chronic dialysis, who may require dialysis coordination, closer monitoring, additional medical consultations, and treatment of complications when they occur. Importantly, the NIS reports hospital charges rather than true costs or reimbursements; therefore, the economic findings should be interpreted as differences in billed hospital charges [18,25,30].

We also observed that the proportion of TKA patients receiving chronic dialysis increased over time. This may reflect a growing willingness to operate on medically complex patients, but it may also be influenced by changes in the overall TKA setting during the study period. In particular, the shift of many lower-risk TKA procedures toward outpatient practice may have increased the relative proportion of medically complex patients captured in an inpatient-only database such as the NIS [31–33]. Accordingly, the increase observed in our study should be interpreted with caution and not necessarily as a direct increase in the true population rate of TKA among individuals receiving chronic dialysis.

The lower coded rate of pulmonary embolism among patients receiving chronic dialysis should be interpreted cautiously. Routine exposure to anticoagulation during hemodialysis may be one possible explanation [34–36]; however, several alternative explanations are also possible. These include differences in perioperative thromboprophylaxis, surveillance bias, competing risk from early severe complications or death, and limitations of administrative coding. In addition, the NIS does not provide information on dialysis modality, timing of dialysis relative to surgery, anticoagulation protocols, or post-discharge thromboembolic events. Therefore, this finding should be considered hypothesis-generating rather than definitive.

The increased coded rate of urinary tract infection should also be interpreted with caution. Some patients receiving chronic dialysis may have reduced urine output or anuria, and the NIS does not provide granular clinical information regarding urine output, catheter use, microbiology, symptoms, or diagnostic criteria. Therefore, urinary tract infection in this analysis reflects an administrative diagnosis code during the index hospitalization rather than a clinically adjudicated outcome.

The markedly increased risks of sepsis and in-hospital mortality deserve special emphasis. Patients receiving chronic dialysis represent a medically fragile population with chronic inflammation, immune dysfunction, and substantial cardiovascular burden [18,37–40]. These biological and clinical factors may help explain the particularly high relative risks observed in our matched analysis. From a practical standpoint, these data reinforce the importance of preoperative counseling, perioperative medical optimization, and close postoperative surveillance in patients receiving chronic dialysis considered for TKA.

### Limitations

This study has several limitations. First, the NIS is an administrative discharge database and is therefore subject to coding inaccuracies, underreporting, and misclassification. Second, the NIS captures inpatient hospitalizations only and does not provide longitudinal follow-up. Therefore, outpatient complications, post-discharge mortality, readmissions, revision surgery, implant survivorship, and long-term functional outcomes could not be evaluated. The results should therefore be interpreted strictly as inpatient outcomes during the index TKA hospitalization.

Third, important dialysis-specific variables were unavailable, including dialysis modality, dialysis duration or vintage, timing of dialysis relative to surgery, dialysis adequacy, vascular access type, anticoagulation exposure, residual urine output, and laboratory values [41–45]. Although ICD-10-CM code Z99.2 was used to identify patients receiving chronic dialysis, the NIS does not provide outpatient lookback information, and misclassification of dialysis status remains possible. Fourth, comorbidities and complications were identified using diagnosis codes recorded during the index hospitalization, and the database does not allow determination of the exact timing, severity, or recency of many conditions.

Fifth, anesthesia type, implant design, fixation method, surgical approach, alignment strategy, intraoperative blood loss, surgeon volume, postoperative rehabilitation protocol, and detailed thromboprophylaxis regimens were not available. Sixth, although propensity score matching improved measured baseline balance, residual confounding from unmeasured variables remains possible. Finally, total hospital charges represent billed hospital charges rather than true costs or reimbursements. Despite these limitations, the large contemporary national sample and matched comparative design provide useful insight into inpatient outcomes after primary TKA among patients receiving chronic dialysis. Although propensity-score matching improved measured baseline balance, post-matching outcome comparisons did not account for matched-set clustering or use robust variance estimation, which may have affected variance estimates and statistical inference. Length of stay and total hospital charges are likely right-skewed variables. Because these outcomes were summarized using means and standard deviations, the reported values should be interpreted as differences in average inpatient resource utilization rather than as a complete characterization of their underlying distributions.

## Conclusions

Chronic dialysis status was associated with significantly worse inpatient outcomes after primary TKA, including higher risks of sepsis, pneumonia, ileus, blood transfusion, intraoperative fracture, and in-hospital mortality, as well as longer LOS and greater hospital charges. Although pulmonary embolism was coded less frequently in the chronic dialysis group, this finding should be interpreted cautiously because of the inpatient-only design and lack of data on anticoagulation, dialysis timing, and post-discharge events. These findings highlight the need for careful patient selection, detailed risk counseling, and multidisciplinary perioperative management when considering TKA in patients receiving chronic dialysis. Future studies should further evaluate long-term outcomes and whether targeted perioperative optimization strategies can improve results in this high-risk population.

## Supporting information

S1 Table Covariate balance before and after 10:1 propensity-score matching. Inpatient outcomes after primary total knee arthroplasty in patients receiving chronic dialysis.
(DOCX)

S2 Table ICD-10-CM and ICD-10-PCS code definitions. (DOCX)
