# Supplementary material for: Inpatient outcomes after primary total knee arthroplasty in patients receiving chronic dialysis: A nationwide propensity score-matched analysis
Source: PLoS One. 2026 Sep 15;21(9):e0358078. doi: 10.1371/journal.pone.0358078 (PMC13577411; doi:10.1371/journal.pone.0358078)
Supplement: Supplementary file 1 [file pone.0358078.s001.docx]

**Supplementary Tables**

Inpatient Outcomes After Primary Total Knee Arthroplasty in Patients Receiving Chronic Dialysis

Note. ICD-10-CM codes are presented in conventional dotted format. In the NIS analytic syntax, decimal points were omitted (for example, Z99.2 was coded as Z992). An asterisk (*) indicates prefix matching, meaning all child codes beginning with that prefix were captured.

**Supplementary Table S1. Covariate balance before and after 10:1 propensity-score matching**

| **Characteristic** | **Before matching SMD** | **After matching SMD** |
| --- | --- | --- |
| Average Age (y) | 0.082 | 0.010 |
| Female sex | 0.298 | 0.002 |
| Robotic surgery | 0.090 | 0.011 |
| Dyslipidemia | 0.140 | 0.016 |
| Chronic anemia | 0.222 | 0.012 |
| Osteoporosis | 0.042 | 0.023 |
| Alcohol abuse | 0.020 | 0.018 |
| Type 2 diabetes | 0.692 | 0.012 |
| Congestive heart failure | 0.384 | 0.013 |
| Chronic lung disease | 0.188 | 0.000 |
| Liver disease | 0.137 | 0.006 |
| History of myocardial infarction | 0.166 | 0.004 |
| History of cerebrovascular accident | 0.201 | 0.007 |
| Obesity | 0.002 | 0.004 |

Abbreviation: SMD, standardized mean difference. An absolute SMD < 0.10 was considered indicative of acceptable balance.
