# Supplementary material for: Inpatient outcomes after primary total knee arthroplasty in patients receiving chronic dialysis: A nationwide propensity score-matched analysis
Source: PLoS One. 2026 Sep 15;21(9):e0358078. doi: 10.1371/journal.pone.0358078 (PMC13577411; doi:10.1371/journal.pone.0358078)
Supplement: Supplementary file 2 [file pone.0358078.s002.docx]

**Supplementary Table S2. ICD-10-CM and ICD-10-PCS code definitions**

| **Domain** | **Variable / definition** | **Code system** | **Code(s) / prefix(es)** | **Operational note** |
| --- | --- | --- | --- | --- |
| Cohort definition | Primary total knee arthroplasty | ICD-10-PCS | 0SRC069; 0SRC06A; 0SRC06Z; 0SRC07Z; 0SRC0J9; 0SRC0JA; 0SRC0JZ; 0SRC0KZ; 0SRC0L9; 0SRC0LA; 0SRC0LZ; 0SRD069; 0SRD06A; 0SRD06Z; 0SRD07Z; 0SRD0J9; 0SRD0JA; 0SRD0JZ; 0SRD0KZ; 0SRD0L9; 0SRD0LA; 0SRD0LZ | Replacement of right or left knee joint with synthetic substitute. |
| Cohort exclusion | Revision / non-primary knee arthroplasty-related procedures | ICD-10-PCS | 0SWC*; 0SWD*; 0SPC*; 0SPD*; 0SUC*; 0SUD* | Revision, removal, or supplement procedures involving the right or left knee joint/prosthesis/synthetic substitute. |
| Cohort exclusion | COVID-19-associated hospitalization | ICD-10-CM | U07.1 | Excluded to reduce confounding from pandemic-related inpatient outcomes. |
| Exposure | Chronic dialysis status | ICD-10-CM | Z99.2 | Dependence on renal dialysis. |
| Surgical / technology covariate | Robotic-assisted surgery | ICD-10-PCS | 8E0Y0CZ; 8E0YXCZ | Robotic-assisted lower-extremity procedure codes. |
| Baseline comorbidity | Dyslipidemia | ICD-10-CM | E78* | Disorders of lipoprotein metabolism and other lipidemias. |
| Baseline comorbidity | Chronic anemia | ICD-10-CM | D64* | Chronic/unspecified anemia coding family used for the chronic anemia flag. |
| Baseline comorbidity | Osteoporosis | ICD-10-CM | M81*; M82* | Osteoporosis coding families. |
| Baseline comorbidity | Alcohol abuse / alcohol-related disorder | ICD-10-CM | F10* | Alcohol-related mental and behavioral disorders. |
| Baseline comorbidity | Type 2 diabetes mellitus | ICD-10-CM | E11* | Type 2 diabetes mellitus, with or without complications. |
| Baseline comorbidity | Congestive heart failure | ICD-10-CM | I50* | Heart failure diagnoses. |
| Baseline comorbidity | Chronic lung disease | ICD-10-CM | J44* | Chronic obstructive pulmonary disease coding family. |
| Baseline comorbidity | Liver disease | ICD-10-CM | K70*; K71*; K72*; K73*; K74*; K75*; K76*; K77* | Alcoholic, toxic, hepatic failure, chronic hepatitis, fibrosis/cirrhosis, inflammatory, vascular, and other liver disease families. |
| Baseline comorbidity | History of myocardial infarction / myocardial infarction diagnosis | ICD-10-CM | I21*; I22* | Acute or subsequent myocardial infarction codes recorded during the index admission. |
| Baseline comorbidity | History of cerebrovascular accident / stroke diagnosis | ICD-10-CM | I60*; I61*; I62*; I63* | Cerebrovascular hemorrhage or infarction codes recorded during the index admission. |
| Baseline comorbidity | Obesity | ICD-10-CM | E66* | Overweight and obesity diagnoses. |
| Outcome | In-hospital mortality | NIS variable | DIED | Death during the index hospitalization. |
| Outcome | Blood loss anemia | ICD-10-CM | D62* | Acute posthemorrhagic anemia. |
| Outcome | Urinary tract infection | ICD-10-CM | N39.0 | Urinary tract infection, site not specified. |
| Outcome | Intraoperative / postprocedural musculoskeletal complication or fracture flag | ICD-10-CM | M96* | Broad intraoperative/postprocedural musculoskeletal complication family used for the intraoperative fracture/surgical complication flag. |
| Outcome | Blood transfusion | ICD-10-PCS | 30233N0; 30233N1 | Transfusion of red blood cells during the index admission. |
| Outcome | Ileus | ICD-10-CM | K56.7* | Ileus coding family. |
| Outcome | Pneumonia | ICD-10-CM | J18.9; J15.9; J22* | Unspecified pneumonia, bacterial pneumonia, or unspecified acute lower respiratory infection categories used in the analysis syntax. |
| Outcome | Sepsis | ICD-10-CM | A40*; A41*; R65* | Streptococcal sepsis, other sepsis, severe sepsis, and septic shock coding families. |
| Outcome | Pulmonary embolism | ICD-10-CM | I26* | Pulmonary embolism coding family. |
| Outcome | Deep vein thrombosis | ICD-10-CM | I82* | Embolism and thrombosis of veins coding family. |

Abbreviations: ICD-10-CM, International Classification of Diseases, Tenth Revision, Clinical Modification; ICD-10-PCS, International Classification of Diseases, Tenth Revision, Procedure Coding System; NIS, Nationwide Inpatient Sample; TKA, total knee arthroplasty.
